# Preamble to the Special Edition Plants and Microgravity

**DOI:** 10.3390/life13051113

**Published:** 2023-04-30

**Authors:** Elizabeth Kordyum, Karl H. Hasenstein

**Affiliations:** 1 Department of Cell Biology and Anatomy, Institute of Botany NASU, Tereschenkivska Str. 2, 01601 Kiev, Ukraine; cellbiol@ukr.net; 2 Biology Department, University of Louisiana at Lafayette, Lafayette, LA 70504, USA

The need to study plant systems in space has a long history and space experiments on plants were recognized for their scientific value and as necessity to provide life support for humans and other non-photosynthetic organisms. Efforts to assess the effect of the space environment, especially weightlessness resulted in essentially continuous plant growth experiments in space beginning with the Salyut space stations through the current advanced possibilities of the Advanced Plant Habitat onboard the International Space Station [1]. The need to either further investigate space experiments or initiate new research programs faces the bottleneck of very limited availability for space flights and therefore often rely on experiments performed on (slow rotating) clinostats or short duration flights on sounding rockets.

Because of the limited time available plant growth studies were initially limited to the seedling stage and therefore focused on fast-growing Arabidopsis cultivars. Early experiments focused on the effect of weightlessness on plant growth and identified the spaceflight syndrome as a complex assembly of stress responses, effects of mechanical unloading and lack of physical conditions such as air movement and water gradients that are common on earth but require technology in space. The special issue “Plants and Microgravity” addresses some of the complex topics that were uncovered as a result of space research.

Many biological phenomena can be investigated only in the absence of the dominant force of gravity. The contribution “Red Light Enhances Plant Adaptation to Spaceflight and Mars *g*-Levels” by Medina et al. illustrates the complex interaction between tropism at different gravitational accelerations (*g*-levels). The ability of red light to alter gene expression and importantly, that not all space experimental conditions are perceived as stress. The possibility to develop targeted approaches that minimize plant stress under uncommon conditions has far-reaching consequences for plant growth in space.

The identification of Earth-gravity as stress factor in the continuum between micro- and hypergravity resulted in the identification of Peptidyl-tRNA Hydrolase II (PTH2) by Hattori et al. as important factor for gravity resistance an essential factor that allows plants to function at various gravitational loads. Gravity adaptations are expressed in all plants are the contribution by Lobachevska et al. illustrates the gravity adaptations in mosses that lead to changes in development and gravity-controlled morphogenesis. Interestingly, in these plants clinorotation and space effects are strikingly similar.

Despite many studies on gravitational effects that focus on the well-known positive and negative gravitropic response of roots and shoots, respectively, the signaling and response mechanism affects not just sedimenting amyloplasts but also includes other cellular compounds and activities. The response of the cell membranes and their effect on metabolism, signal transduction, and adaptive responses of microdomains (lipid rafts) is discussed by Kordyum et al.

Technical challenges for especially short-duration experiments resulted in sophisticated and robust technologies such as the Kennedy Space Center developed fixation tubes that have proven themselves many times. Haveman at al. describe using the fixation tubes as miniature growth chambers that can be fixed in RNA-Later and processed for down-stream investigations such as RNA sequencing.

In contrast to studies using dicotyledonous plants, the contribution by Su et al. focuses on grass plants and the morphological and transcriptome response to space flight. The accession specific responses are indicative of many different strategies that plants can exploit to adapt to growth in space and gravity conditions other than Earth’s evolutionarily imprinted acceleration level.

The papers in the special edition provide a thorough window into the many research directions that can be pursued to improve growth of diverse plants as part of the biological life support systems and the acknowledged psychological benefits for humans under confined life conditions on future outposts on Moon or Mars.
